# Fecal Microbiota and Its Correlation With Fatty Acids and Free Amino Acids Metabolism in Piglets After a *Lactobacillus* Strain Oral Administration

**DOI:** 10.3389/fmicb.2019.00785

**Published:** 2019-04-16

**Authors:** Dongyan Zhang, Hui Liu, Sixin Wang, Wei Zhang, Jing Wang, Hongwu Tian, Yamin Wang, Haifeng Ji

**Affiliations:** ^1^Institute of Animal Husbandry and Veterinary Medicine, Beijing Academy of Agriculture and Forestry Sciences, Beijing, China; ^2^Beijing Research Center of Intelligent Equipment for Agriculture, Beijing, China

**Keywords:** fecal microbiota, *Lactobacillus reuteri*, piglets, fatty acids, free amino acids, correlation analysis

## Abstract

*Lactobacillus* has a positive effect on the host intestinal microbiota. In piglets, dietary supplementation with *Lactobacillus* affects general health and plays an important role in nutrient digestion and fermentation. However, this association requires further investigation. Here, we studied newborn piglets from 12 litters. The nursed piglets were given a creep feed beginning on day 10 *post-partum* and weaned at day 30. Piglets were fed either a control basic diet or a diet including supplementation with *Lactobacillus reuteri* ZLR003 at 6.0 × 10^6^ CFU/g feed. At day 30 and 60, feces samples were taken and used for sequencing of the V3-V4 hypervariable region of the 16S rRNA gene. At day 60, feces samples and serum samples were also taken and used to measure the short chain fatty acids (SCFAs) and to detect long chain fatty acids (LCFAs) and free amino acids (FAAs), respectively. The results revealed that *L. reuteri* ZLR003 could improve piglet fecal microbiota composition, especially at the end of weaned period. The concentrations of lactic acid and butyric acid in feces were higher, and acetic acid concentration was lower in the *L. reuteri* ZLR003 group compared with the control group (*P* < 0.05). The serum polyunsaturated fatty acids C18:2n6c, C18:3n3, C20:4n6, and C22:6n3 were significantly higher (*P* < 0.05), as were the serum FAAs Gly, Ala, Val, Iso, Asn, Asp, Glu, Met, Phe, and Leu (*P* < 0.05), in the *L. reuteri* group compared with the control group. A correlation analysis revealed that the genera *Ruminococcaceae*_UCG-010 and *Ruminococcaceae*_UCG-014 had a negative correlation with the SCFAs content in feces, the genus *Prevotella*_9 had a higher positive correlation with C18:2n6c, and the genera *Megasphaera* and *Mitsuokella* had a more positive significant effect on the serum FAAs content in weaned piglets in the *L. reuteri* ZLR003 group compared with the control group. In conclusion, *L. reuteri* ZLR003 influenced the fecal microbiota composition of piglets, and its effects were related to the metabolism of SCFAs, LCFAs, and FAAs. Our findings will help facilitate the application of *Lactobacillus* strains in pig production.

## Introduction

*Lactobacillus*, an essential member of the normal microbiota, can improve the intestinal microbial balance with beneficial effects when administered in adequate amounts (Bogovic et al., 2016; Simpson et al., 2018). *Lactobacillus* has been widely employed as a supplement in foods as well as in farming and medicine (Hill et al., 2014). In fact, the *Lactobacillus* market has maintained a growth rate of 15–18% in recent years in China^1^. There is increased acceptance of the application of *Lactobacillus* strains in animal feeds to maintain health, and animal studies have shown the beneficial probiotic effects. However, the mechanism of action of *Lactobacillus* remains to be elucidated.

During the weaning period, piglets are subjected to sudden dietary, social, and environmental changes, and the microbiota are sharply changed, generally resulting in a decrease in bacteria of the *Lactobacillus* group and an increase in pathogenic bacteria, mainly leading to colibacillosis resulting in diarrhea, poor appetite, lower feed intake, and growth retardation (Isaacson and Kim, 2012; Schokker et al., 2015). Previous studies on the microbiota in weaning piglets suggested that there was a large reduction in alpha diversity during the weaning period, and that this was reduced further after treatment with antibiotics (Pie et al., 2004; Tao et al., 2015; Dou et al., 2017). Therefore, weaning has been associated with a “disrupted state” for the microbiota that is referred to as “dysbiosis,” and weaning stress in piglets is always the major cause of economic loss for pig farmers (Wilson et al., 1989; Lalles et al., 2007).

Metagenomics and related methods have led to significant advances in our understanding of the gut microbiome. Supplementation with *Lactobacillus* modulates the gut microbial profile and thereby affects microbial metabolite production, which results in improved gut health (Chiang et al., 2015; Dowarah et al., 2017a). Researchers have also reported that *Lactobacillus* strains could be effective at enhancing intestinal barrier function, which restricts the colonization of pathogenic bacteria to the intestinal mucosa, as evidenced by better absorption of nutrients, enabling better sustainability of the piglets (Abrahao et al., 2004).

Previously, *Lactobacillus reuteri* ZLR003 was isolated from the caecum mucosa of a weaned piglet. Our previous work showed that this strain affected the gut microbiota composition in the jejunum, colon, and cecum of weaned piglets (Zhang et al., 2016). In addition, the results of Kyoto Encyclopedia of Genes and Genomes (KEGG) pathway analysis by RNA-seq in the jejunum tissue showed that the expression of genes related to arachidonic acid metabolism and linoleic acid metabolism, glutathione metabolism, glycine, and serine and threonine metabolism were improved after treatment with *L. reuteri* ZLR003 compared with the control group (Zhang D.Y. et al., 2017). We hypothesized that a shift in microbiota might be associated with a shift in microbiota products and blood metabolites, although the possible link was still unclear. Thus, in our present study, we analyzed changes in the fecal microbiota composition during the nursed and weaned periods following supplementation with *L. reuteri* ZLR003 and evaluated the underlying association with feces short chain fatty acids (SCFAs), serum long chain fatty acids (LCFAs), and serum free amino acids (FAAs) affected by the microbiota in weaned piglets.

## Materials and Methods

### *Lactobacillus* Strain

*Lactobacillus reuteri* ZLR003 was isolated from the cecum mucosa of a healthy weaned piglet in our laboratory. The strain was identified by the China Center of Industrial Culture Collection (Beijing, China), and had been preserved at the China General Microbiological Culture Collection Center (CGMCC No. 11530). Overnight cultures of *L. reuteri* ZLR003 grown in MRS broth at 37°C were centrifuged at 6738 × *g* for 10 min. The harvested cells were then resuspended in 20 L of protective agent and freeze-dried in a pilot-scale apparatus (CHRIST, Epsilon 2-60, Germany, Osterode) for 23 h. The resulting freeze-dried powder was vacuum-packed and stored at -20°C.

### Animals and Treatment Procedure

The experiments were carried out in a commercial farrow-to-finish pig farm in Beijing (Xingwangyuan Co. Ltd., Beijing, China). Landrace × Large White newborn piglets with a similar initial weight (14.5 ± 0.25 kg of piglets/litter, both sexes) from 12 sows were used in this experiment (*n* = 6 per group). Each crate was equipped with a heat lamp for newborn piglets. The nursed piglets had a single feeder and a nipple drinker, and beginning at *post-partum* day 10, they were given access to a creep feed, the contents of which differed for the control and *L. reuteri* ZLR003 groups. All the piglets were weaned at 30 days, at which time the piglets were removed to a controlled environment with stainless steel beds and were allowed *ad libitum* access to feed and water through a feeder and nipple drinker. The weaned piglets in the two treatment groups were kept separate and provided with control and *L. reuteri* ZLR003 feed. Each treatment group had 6 replicate pens with 10–11 piglets per pen. Throughout the experimental period, the diets of the nursed and weaned piglets were formulated to meet the standard growth requirements for piglets weighing 3–8 kg or weighing 8–20 kg (Feeding Standard of Swine, 2004; Table 1). The control group received a basic diet (free of probiotics and antibiotics), and the *L. reuteri* ZLR003 group was administered the basic diet supplemented with freeze-dried *L. reuteri* ZLR003 (6.0 × 10^6^ CFU/g feed). The two kinds of diet were prepared once every 5 days. The full experimental period lasted 60 days.

**Table 1:** Ingredients and composition of the basal diet for piglets.

Ingredients (g/kg)	3–8 kg	8–20 kg
corn	240.0	600.0
Extruded corn	250.0	0
Full fat soybeans	130.0	50.0
Extruded soybeans	120.0	70.5
Soybean	0	130.0
Fish meal	55.0	20.0
Whey	150.0	40.0
Wheat bran	0	40.0
Soybean oil	15.0	10.0
Dicalcium phosphate	10.0	10.0
Calcium carbonate	6.0	8.0
L-Lysine-HCL,780 g/kg	6.0	5.0
DL-Methionine, 980 g/kg	0.6	0.5
L-Threonine, 980 g/kg	2.0	1.2
L-Tryptophan, 980 g/kg	1.4	0.8
Sodium chloride	4.0	4.0
Vitamin-mineral premix	10	10.0
Chemical composition		
Digestible energy^a^, MJ/kg	14.15	13.75
Crude protein^b^	19.85	18.55
Lysine^a^	14.5	13.2
Methionine + cysteine^a^	9.05	8.45
Threonine^a^	9.85	9.75
Tryptophan^a^	3.5	3.14
Calcium^b^, g/kg	7.2	6.8
Total phosphorus^b^, g/kg	5.6	5.4


*^*a*^Each kg of complete feed contains: vitamin A, 11,000 IU; vitamin D_*3*_, 2,800 IU; vitamin E, 36 mg; menadione, 2.5 mg; vitamin B_*1*_, 2.5 mg; vitamin B_*2*_, 6.6 mg; vitamin B_*6*_, 3.0 mg; vitamin B_*12*_, 0.025 mg; niacin, 25 mg; pantothenic acid, 13 mg; biotin, 0.2mg; Mn, 55 mg; Fe, 120 mg; Zn, 100 mg; Cu, 12 mg; I, 0.50 mg; and Se, 0.30 mg. ^*a*^Calculated nutrient levels. ^*b*^Measured nutrient levels.*

### Sample Collection

Fresh fecal samples from 24 (*n* = 12 per group) and 36 (*n* = 18 per group) piglets were randomly collected at day 30 and 60 of the trial, respectively. The samples from three piglets were combined into one sample, such that a total of 8 (*n* = 4 per group) and 12 (*n* = 6 per group) process samples were collected at days 30 and 60, respectively. All of the fecal samples were collected in sterile containers, snap-frozen in liquid nitrogen, and stored at -80°C. The fecal samples from day 30 were used to analyze the microbiota composition, and the fecal samples from day 60 were used to analyze both the microbiota composition and the SCFAs concentration.

Additionally, on day 60, 5-mL blood samples were collected from 16 selected (*n* = 8 per group) piglets before morning feeding at the end of the trial by precava venipuncture. Blood was centrifuged at 1, 238 × *g* for 10 min at 4°C, and the resulting serum was frozen at -20°C for use in the subsequent analysis of the LCFAs and FAAs content. The experimental design is shown in Supplementary Table S1.

### DNA Extraction, PCR Amplification, and Sequencing

Total bacterial DNA was extracted from the collected feces samples using the EZNA^®^ Stool DNA Kit (Omega Bio-tek, Norcross, GA, United States) according to the manufacturer’s protocols. The DNA quality was determined using agarose gel electrophoresis and a NanoDrop 8000 spectrophotometer (Thermo Fisher Scientific, Scoresby, Australia). Amplification of the V3-V4 region of the bacterial 16S ribosomal RNA gene was performed with the following PCR cycling conditions: an initial denaturation at 95°C for 3 min; followed by 27 cycles of 95°C for 30 s, 55°C for 30 s, and 72°C for 45 s; and finished with a final extension at 72°C for 10 min. The primers 338F (5’-ACTCCTACGGGAGGCAGCA-3’) and 806R (5’-GGACTACHVGGGTWTCTAAT-3’) were designed with a sequence attached eight-base barcode that was unique to each sample. The PCR was carried out in triplicate using 4 μL of 5 × FastPfu buffer, 2 μL of 2.5 mM dNTPs, 0.8 μL of each primer (5 μM), 0.4 μL of FastPfu polymerase, and 10 ng of template DNA in a final volume of 20 μL.

The PCR products were extracted from 2% agarose gels, purified using the AxyPrep DNA Gel Extraction Kit (Axygen Biosciences, Union City, CA, United States) according to the manufacturer’s instructions and quantified using QuantiFluor^TM^-ST (Promega, Madison, WI, United States). Purified amplicons were pooled in equimolar amounts and paired-end sequenced (2 × 250) on an Illumina MiSeq platform according to standard protocols.

### High-Throughput 16S rRNA Sequencing and Bioinformatics Analysis

Raw fastq files were demultiplexed and quality filtered using QIIME (version 1.17) according to the following criteria: (i) the 300-bp reads were truncated at any site receiving an average quality score of <20 over a 50-bp sliding window, discarding the truncated reads that were shorter than 50 bp; (ii) exact barcode matches, two- nucleotide primer mismatches, and reads containing ambiguous characters were removed; and (iii) only sequences that overlapped more than 10 bp were assembled. Reads that could not be assembled were discarded. Operational units (OTUs) were clustered using UPARSE (version 7.1^2^) at a 97% similarity level, and chimeric sequences were identified and removed using UCHIME. Taxonomic classification of phylotypes was determined using the Ribosomal Database Project Classifier^3^ against the Silva (SSU115) 16S rRNA database at a 70% confidence threshold (Amato et al., 2013).

### Analysis of the Fecal SCFAs Composition in Weaned Piglets

The fecal SCFAs composition of weaned piglets was determined by gas chromatography (6890 series, Agilent Technologies, Wilmington, DE, United States) according to the procedures of Pierce et al. (2005) with slight modifications. Standards of lactic acid, formic acid, acetic acid, propionic acid, isobutyric acid, butyric acid, isovaleric acid, pentoic acid, and 1, 3-butanediol (as the internal standard) were purchased from Sigma Aldrich (Sigma-Aldrich, St Louis, MO, United States) and were of HPLC grade with >99% purity. Before the SCFAs analysis, a 1-g fecal sample was homogenized in 10 mL of distilled water and centrifuged at 12, 000 × *g* for 10 min, after which the resulting supernatant was filtered through a 0.22-μm syringe filter. Aliquots of 1 μL were injected into a capillary column (60 m × 250 μm × 0.25 μm, DB-23, Agilent Technologies) with cyanopropyl methyl silicone as the stationary phase. The column temperature was programmed with a 1:20 split. Injector and detector temperatures were each maintained at 240°C. Nitrogen was the carrier gas at a flow rate of 2.0 mL/min.

### Analysis of the Serum LCFAs Composition in Weaned Piglets

The serum LCFAs composition was determined by gas chromatography (6890 series, Agilent Technologies) according to the procedures of Sukhija and Palmquist (1988) with slight modifications. The standards of 20 LCFAs and internal standard (C11:0) (Sigma-Aldrich, St Louis, MO, United States; HPLC grade, >99%) were used in the experiment. A 1-mL sample of serum was added into 4 mL of a mixed solution of ethyl chloride and methanol, after which a 1-mL internal standard hexane solution was added to the sample. The tube was subsequently incubated in a hot water bath at 80°C for 2 h, then cooled to room temperature and combined with 5 mL of a 100 g/L potassium chloride solution. Aliquots of 1 μL were injected into a capillary column (60 m × 250 μm × 0.25 μm, DB-23, Agilent Technologies) with cyanopropyl methyl silicone as the stationary phase. The column temperature was programmed with a 1:20 split. Injector and detector temperatures were maintained at 260°C and 270°C, respectively. Nitrogen was the carrier gas at a flow rate of 2.0 mL/min.

### Analysis of Serum FAAs in Weaned Piglets

The serum FAAs composition was determined by HPLC-MS/MS (LC20AD-API 3200MD TRAP, Shimadzu, Japan) with post-column ninhydrin derivatization and the use of a fluorescence detector, as described by Fekkes (1996) with slight modifications. Amino acid standards and internal standard (L -theanine) were also obtained from Sigma Aldrich (Sigma-Aldrich, St Louis, MO, United States; HPLC grade, >98%). Aliquots of 3 μL were injected into a capillary column (15 cm × 4.6 mm × 5 μm, MSLab 45+AA-C18, Agilent Technologies) with cyanopropyl methyl silicone as the stationary phase. The column temperature was 50°C, and the flow rate was 1 mL/min.

### Statistical Analyses

Statistical analyses of the alpha-diversity indices, fatty acids compositions, and FAAs compositions were tested using the general linear models (GLM) procedure in SAS software. Differences among means were tested using Tukey’s test, and differences were considered significant at *P* < 0.05. Correlations between bacterial taxa and fatty acids or FAAs were calculated by non-parametric Spearman’s rank correlation analysis using JMP version 10 (SAS Inst. Inc., Cary, NC, United States). The microbiota data were analyzed on the free online platform of Majorbio I-Sanger Cloud Platform ^4^.

## Results

### DNA Sequence Data

For nursed (day 30) and weaned (day 60) piglets, respectively, a total of 449,779 and 2,979,655 paired-end reads comprising 270,766,958 and 917,047,670 bp were generated from the raw data, and 449,779 and 1,489,418 valid sequences remained after chimeras were filtered out and low-quality sequences were removed. Among the high-quality sequences, about 99.98% were between 421 and 460 bp for the two time periods, with an average of 439.91 bp (day 30) and 440.23 bp (day 60). The rarefaction curves suggested that this sequencing depth was sufficient to cover the microbial diversity of each sample (Supplementary Figures S1, S2).

### Alpha Diversity of the Fecal Microbiota

Sequence information and calculated microbial diversity indexes of the samples are shown in Table 2. The index of Good’s coverage was >99.5% for all samples, suggesting that the most representative bacterial phylotypes in the fecal samples were obtained by the analysis. Although the *L. reuteri* ZLR003 group exhibited trends toward a higher diversity compared with the control group for both nursed (day 30) and weaned (day 60) piglets according to the ACE, Chao1, Shannon, and Simpson indices, these differences did not reach statistical significance (*P* > 0.05).

**Table 2:** Microbial alpha diversity indices of different treatment groups.

Treatments	Abundance		Coverage	Diversity	
			
	ACE	Chao1		Shannon	Simpson
Nursed piglets					
Control	478.7	513.1	0.9984	3.90	0.053
*Lactobacillus reuteri* ZLR003	482.3	515.3	0.9985	4.04	0.052
SEM	15.48	15.69		1.755	0.00624
*P*-value	0.544	0.382		0.553	0.421
Weaned piglets					
Control	587.9	585.2	0.9955	4.66	0.0258
*L. reuteri* ZLR003	589.8	588.2	0.9955	4.70	0.0268
SEM	7.47	6.59		0.039	0.00133
*P*-value	0.322	0.354		0.447	0.582


*The ACE, Chao1, Shannon and Simpson indexes are presented for a similarity of 0.97 between reads.*

### Shifts in the Piglets Microbiota From the Nursed to the Weaned Period

**FIGURE 1:**
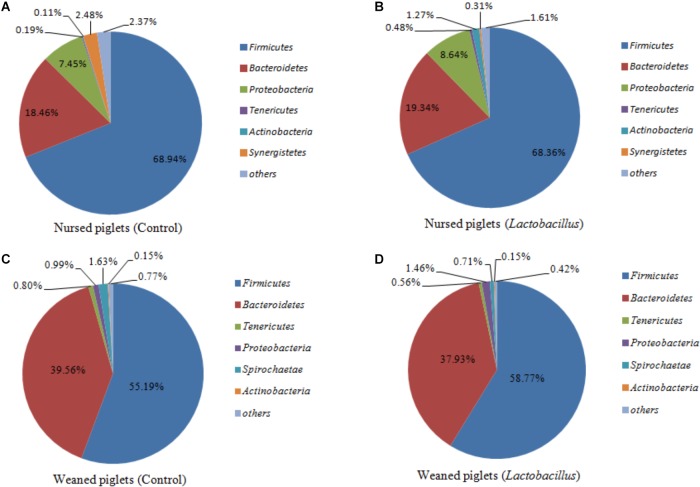
**(A)** Nursed piglets in control group, **(B)** Nursed piglets in *L. reuteri* ZLR003 group, **(C)** Weaned piglets in control group, and **(D)** Weaned piglets in *L. reuteri* ZLR003 group. The fecal microbiota composition of nursed and weaned piglets at the phylum level.

*Firmicutes, Bacteroidetes, Proteobacteria*, and *Actinobacteria* were the dominant phyla in nursed (day 30) and weaned (day 60) piglets from both treatment groups, representing more than 97% of taxa detected in the fecal samples. The abundances of *Firmicutes* (68.65% vs. 56.68%, *P* = 0.019) and *Proteobacteria* (8.05% vs. 1.23%, *P* = 0.009) were significantly decreased, while the abundance of *Bacteroidetes* (18.90% vs. 38.75%, *P* = 0.002) was significantly increased in piglets at the end of the weaned period compared with the nursed period (Figure 1). At the genus level, *Lachnospiraceae* (2.01% vs. 2.74%, *P* = 0.237), *Prevotellaceae* (1.46% vs. 8.47%, *P* = 0.001), *Prevotella* (6.66% vs. 22.70%, *P* = 0.002), *Streptococcus* (0.031% vs. 5.77%, *P* < 0.001), and *Lactobacillus* (3.41% vs. 3.57%, *P* = 0.147) were all more abundant in weaned piglets than in nursed piglets.

### Effects of Dietary Supplementation With *L. reuteri* ZLR003 on Fecal Microbiota Composition for Nursed and Weaned Piglets

**FIGURE 2:**
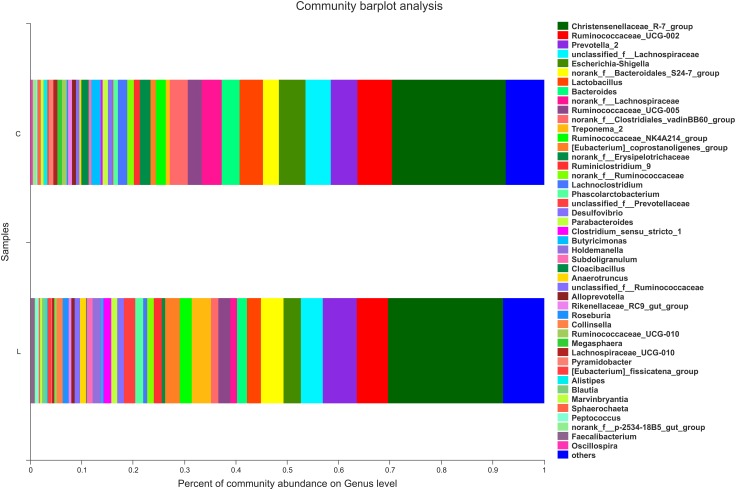
The fecal microbiota composition of nursed piglets at the genus level. C, control group; L, *L. reuteri* ZLR003 group.

For nursed (day 30) piglets, there were no significant differences in microbiota composition at the phylum level between the control and *L. reuteri* ZLR003 groups. However, at the genus level, the abundances of the genera *Prevotellaceae* (0.51% vs. 2.42%, *P* = 0.002) and *Prevotella* (5.87% vs. 7.47%, *P* = 0.015) were significantly higher in the *L. reuteri* ZLR003 group compared with the control group for the nursed piglets (Figure 2).

**FIGURE 3:**
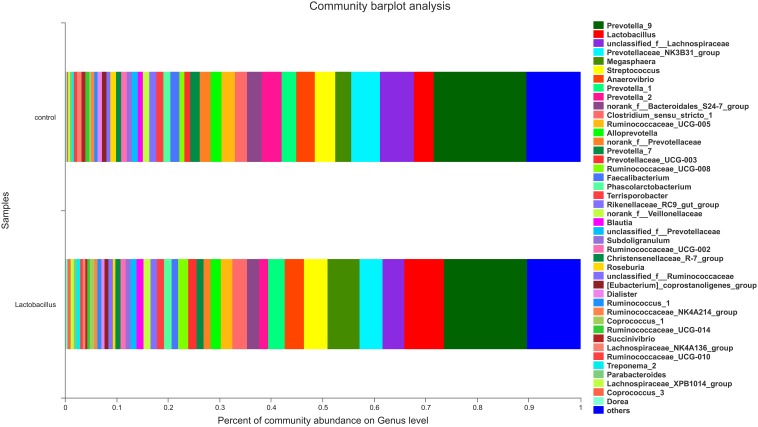
The fecal microbiota composition of weaned piglets at the genus level. C, control group; L, *L. reuteri* ZLR003 group.

In contrast, there was an obvious effect on the microbiota structure of piglets after dietary supplementation with *L. reuteri* ZLR003 at the end of weaned period (day 60) (Figure 1). At the phylum level, the abundance of *Firmicutes* was higher in the *L. reuteri* ZLR003 group than in the control group (58.77% vs. 55.19%, *P* = 0.042), whereas the abundances of *Bacteroidetes* (37.93% vs. 39.56%, *P* = 0.035) and *Spirochaetae* (0.71% vs. 1.63%, *P* = 0.016) were significantly lower in the *L. reuteri* ZLR003 group compared with the control group. In addition, at the genus level, *Prevotella*_9 (12.48% vs. 11.30%, *P* = 0.021), *Megasphaera* (9.09% vs. 3.70%, *P* = 0.016), *Lactobacillus* (3.80% vs. 2.56%, *P* = 0.016), *Selenomonas* (4.67% vs. 2.56%, *P* = 0.020), and *Mitsuokella* (0.683% vs. 0.175%, *P* = 0.025) were significantly more abundant in the *L. reuteri* ZLR003 group compared with the control group. Furthermore, the genera *Streptococcus* (4.18% vs. 7.36%, *P* = 0.022), *Treponema*-2 belonging to *Spirochaetae* (0.62% vs. 1.59%, *P* = 0.029), *Ruminococcaceae*_UCG-014 (1.35% vs. 1.61%, *P* = 0.039), *Ruminococcaceae*_UCG-010 (0.38% vs. 0.47%, *P* = 0.043), and *Clostridium_sensu_stricto* genus (1.11% vs. 2.67%, *P* = 0.042) were all significantly less abundant in the *L. reuteri* ZLR003 group compared with the control group (Figure 3). Moreover, there was no difference in the abundance of the *Lachnospiraceae* genus (12.14% vs. 11.87%, *P* = 0.814) between the two treatment groups.

### Analysis of the Fecal SCFAs Composition in Weaned Piglets

The fecal SCFAs composition in weaned piglets from the two groups is shown in Table 3. The concentrations of lactic acid and butyric acid were higher and that of acetic acid was lower in the *L. reuteri* ZLR003 group compared with the control group (*P* < 0.05).

**Table 3:** Effects of dietary supplementation with *L. reuteri* ZLR003 on fecal SCFAs composition in weaned piglets.

Fatty acid composition (mg/kg)	Control	*L. reuteri*	*P-value*	SEM^A^
Lactic acid	617.90	823.27	0.013	43.001
Formic acid	30.58	34.98	0.232	1.767
Acetic acid	4054.85	3910.10	0.022	32.811
Propionic acid	2517.07	2528.77	0.232	56.277
Isobutyric acid	219.42	227.333	0.560	8.361
Butyric acid	1578.10	1686.17	0.019	24.51
Isovaleric acid	231.60	238.42	0.123	6.359
Pentoic acid	405.07	453.73	0.145	16.117


*^*A*^SEM, standard error of the mean.*

### Analysis of the Serum LCFAs Composition in Weaned Piglets

The LCFAs profiles in serum, including saturated fatty acids (SFAs), monounsaturated fatty acid (MUFAs), and polyunsaturated fatty acids (PUFAs), were determined for weaned piglets in the two treatment groups. The PUFAs C18:2n6c, C18:3n3, C20:4n6, and C22:6n3 were significantly more abundant in the *L. reuteri* group compared with the control group (*P* < 0.05) (Table 4), while there were no differences in the SFAs and MUFAs serum compositions between the control and *L. reuteri* groups (*P* > 0.05).

**Table 4:** Effects of dietary supplementation with *L. reuteri* ZLR003 on serum LCFAs composition in weaned piglets.

Fatty acid composition (ug/mL)	Control	*L. reuter*i	*P*-value	SEM^A^
Saturated fatty acids (SFAs)				
C12:0	3.17	3.59	0.364	0.217
C14:0	0.95	0.92	0.858	0.077
C15:0	3.31	2.68	0.400	0.362
C16:0	125.41	132.35	0.641	7.365
C17:0	12.37	11.44	0.710	1.169
C18:0	79.76	76.31	0.686	4.034
C20:0	2.33	2.89	0.118	0.181
C21:0	3.901	3.866	0.519	0.197
C22:0	0.53	0.62	0.162	0.039
C24:0	7.13	7.17	0.564	0.334
Monounsaturated fatty acids (MUFAs)				
C16:1	4.23	4.59	0.688	0.411
C18:1n9c	97.67	93.74	0.400	2.667
C20:1	0.96	0.98	0.096	0.091
C24:1	4.52	4.43	0.137	0.081
Polyunsaturated fatty acids (PUFAs)				
C18:2n6c	140.06	160.61	0.042	5.561
C18:3n3	3.47	5.26	0.049	3.472
C20:3n6	2.92	3.69	0.15	0.276
C20:4n6	44.98	47.33	0.035	0.573
C20:5n3	0.93	0.94	0.347	0.121
C22:6n3	3.58	3.75	0.035	0.050


*^A^SEM, standard error of the mean.*

### Analysis of the Serum FAAs in Weaned Piglets

The content of serum FAAs, including glycine (Gly), alanine (Ala), valine (Val), isoleucine (Iso), asparagine (Asn), asparaginic acid (Asp), glutamic acid (Glu), methionine (Met), phenylalanine (Phe), arginine (Arg), lysine (Lys), and leucine (Leu), in weaned piglets was significantly higher in the *L. reuteri* group compared with the control group (*P* < 0.05) (Table 5).

**Table 5:** Effects of dietary supplementation with *L. reuteri* ZLR003 on serum FAAs composition in weaned piglets.

Free amino acids (ug/mL)	Control	*L. reuteri*	*P-value*	SEM^A^
Glycin (Gly)	44.012	49.418	0.044	1.431
Alanine (Ala)	40.396	45.068	0.034	1.108
Serine (Ser)	14.578	17.695	0.050	0.809
Proline (Pro)	26.779	30.023	0.055	0.810
Valine (Val)	17.924	21.467	0.004	0.678
Threonine (Thr)	14.524	15.671	0.667	1.246
Cysteine (Cys)	4.711	5.529	0.359	0.419
Isoleucine (Iso)	8.851	12.479	0.001	0.613
Asparagine (Asn)	8.665	10.996	0.004	0.461
Asparaginic acid (Asp)	2.516	4.164	0.002	0.296
Glutamine (Gln)	51.423	44.027	0.159	2.638
Glutamic acid (Glu)	19.736	31.743	0.016	2.604
Methionine (Met)	4.583	6.886	0.002	0.417
Histidine (His)	13.101	13.876	0.362	0.398
Phenylalanine (Phe)	14.206	16.436	0.013	0.476
Arginine (Arg)	42.455	53.632	0.019	2.444
Tryptophan (Try)	4.161	5.310	0.255	0.020
Lysine (Lys)	12.073	19.122	0.003	1.333
Tyrosine (Tyr)	14.959	17.014	0.303	0.957
Leucine (Leu)	15.859	21.368	0.001	0.915


*^*A*^SEM, standard error of the mean.*

### The SCFAs, LCFAs, and FAAs Affected by Microbiota in Weaned Piglets

**FIGURE 4:**
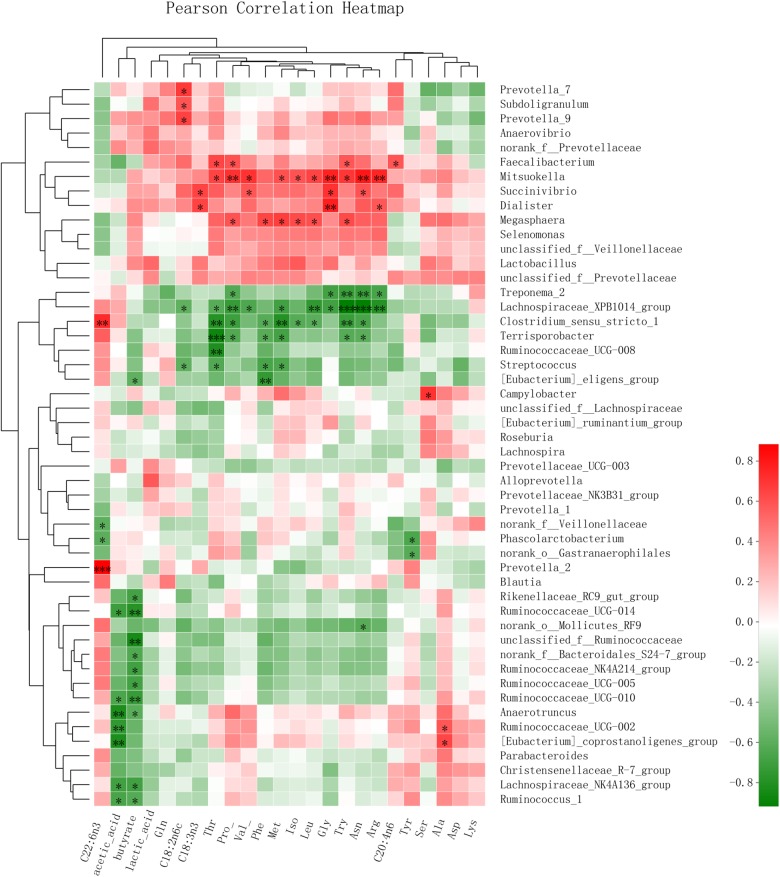
Pearson correlation heatmap of the SCFAs in feces and the LCFAs and FAAs in serum, affected by microbiota changes in weaned piglets. ^∗^0.01 < *P* ≤ 0.05, ^∗∗^0.001 < *P* ≤ 0.01, ^∗∗∗^*P* ≤ 0.001.

A Pearson correlation heatmap (Figure 4) was used to analyze the association of the microbiota with the SCFAs in feces and with the LCFAs and FAAs in serum. The genera *Ruminococcaceae_*UCG_1 (Pearson value, *r* = -0.660, *P* = 0.020; *r* = -0.618, *P* = 0.032), *Ruminococcaceae*_UCG-014 (*r* = -0.702, *P* = 0.011; *r* = -0.782, *P* = 0.003), and *Ruminococcaceae*_UCG-010 (*r* = -0.653, *P* = 0.021; *r* = -0.725, *P* = 0.008) all showed a negative correlation with the content of acetic acid and butyric acid in feces, respectively. (*Eubacterium*)_*coprostanoligenes*_*group* (*r* = -0.817, *P* = 0.001) showed a negative correlation with acetic acid. Additionally, the genera *Prevotella*_7 (*r* = 0.652, *P* = 0.022) and *Prevotella*_9 (*r* = 0.687, *P* = 0.014) had a positive correlation with C18:2n6c. Furthermore, *Mitsuokella* was positively correlated with Gly (*r* = 0.771, *P* = 0.003), Pro (*r* = 0.713, *P* = 0.009), Val (*r* = 0.711, *P* = 0.010), Thr (*r* = 0.649, *P* = 0.022), Iso (*r* = 0.591, *P* = 0.043), Asn (*r* = 0.801, *P* = 0.002), Met (*r* = 0.602, *P* = 0.038), Arg (*r* = 0.756, *P* = 0.004), Try (*r* = 0.694, *P* = 0.012), and Leu (*r* = 0.649, *P* = 0.022), and *Megasphaera* was positively correlated with Pro (*r* = 0.593, *P* = 0.042), Iso (*r* = 0.620, *P* = 0.031), Met (*r* = 0.667, *P* = 0.018), Phe (*r* = 0.643, *P* = 0.024), and Try (*r* = 0.621, *P* = 0.031). The genus *Dialister* was positively correlated with Gly (*r* = 0.776, *P* = 0.003), and the genus *Campylobacter* was positively correlated with Ser (*r* = 0.708, *P* = 0.010). Additionally, the genus *Lachnospiraceae*_XPB1014_group showed a negative correlation with the content of Pro (*r* = -0.727, *P* = 0.007), Thr (*r* = -0.606, *P* = 0.037), Asn (*r* = -0.914, *P* < 0.001), and Try (*r* = -0.643, *P* < 0.001). *Streptococcus* showed a negative correlation with the content of Thr (*r* = -0.643, *P* = 0.029), Met (*r* = -0.643, *P* = 0.024), Try (*r* = -0.918, *P* = 0.019), and Leu (*r* = -0.749, *P* = 0.019).

## Discussion

Piglets are thought to be bacteria-free prior to birth. After birth, the flexible distribution of intestinal microbes leads to differences in microbiota composition, which is influenced by factors such as the porcine species, age, feed, and husbandry (Zhao et al., 2015). Reports have suggested that dietary supplementation with *Lactobacillus* helps in the early development of a stable gut microbiota in neonatal piglets and reduces the number of potential entero-pathogens, such as *Escherichia coli* and *Clostridia*, in neonatal weaned piglets by improving intestinal health and preventing diarrhea (Liu et al., 2014; Dowarah et al., 2017b).

High-throughput next-generation sequencing of 16S rRNA genes has provided abundant information on microbiota composition and has contributed to our understanding of the function of the intestinal microbiota in animal health. Previous research showed that *Firmicutes* and *Bacteroidetes* are the most abundant phyla in the fecal microbiota of pigs (Looft et al., 2014). The results of our present study similarly show that *Firmicutes* and *Bacteroidetes* were the dominant phyla in the feces of both treatment groups of nursed and weaned piglets. However, the total proportion of *Firmicutes* and *Bacteroidetes* was increased from 86 to 94% from the nursed to the weaned period, with a higher proportion of the *Proteobacteria* phylum (about 7%) observed during the nursed period. The proportion of *Firmicutes* present at the end of the nursed period in our study is consistent with the results published by Zhao et al. (2015). Who reported the presence of 73.0% of *Firmicutes* among 30-day-old piglets. The abundance of the *Firmicutes* phylum was decreased while that of the *Bacteroidetes* phylum was increased for weaned piglets compared with nursed piglets in both the control and *L. reuteri* ZLR003 groups. At the genus level, *Prevotella* was clearly increased from 7 to 31% between the nursed and weaned periods for piglets. Arumugam et al. (2011) reported that the *Bacteroidetes* phylum is important for the degradation of carbohydrates. Furthermore, *Prevotella spp*. has been reported to play a role in carbohydrate metabolism and was found to dominate the swine fecal metagenome (Surana and Kasper, 2017). Thus, we hypothesize that the changes in the abundance of the *Bacteroidetes* phylum and *Prevotella* genus are related to changes in the diet of piglets from the nursed to weaned period.

Our data reveal that the microbiota composition profile in weaned piglets supplemented with *L. reuteri* ZLR003 was distinct from that of weaned control piglets. Specifically, at the genus level, *Selenomonas, Mitsuokella, Megasphaera*, and *Lactobacillus*, all of which belong to the *Firmicutes* phylum, were more abundant in the *L. reuteri* ZLR003 group compared with the control group. We analyzed carbohydrate fermentation in accordance with Bergey’s ManuaCl of Determinative Bacteriology (2004) and found that *Selenomonas* (acetate and propionate), *Mitsuokella* (acetate), *Megasphaera* (lactate), and *Lactobacillus* (lactate) were able to produce SCFAs, including lactate, acetate, and propionate. Furthermore, our results indicate that *L. reuteri* ZLR003 dietary supplementation affected the SCFAs content of piglets, based on comparison with the control group. However, there was no significant difference in the abundance of *Lachnospiraceae* between the *L. reuteri* and control groups. Furthermore, the abundance of the *Spirochaetae* phylum was significantly lower in the *L. reuteri* ZLR003 group compared with the control group, as was the abundance of the *Treponema* genus. Our previous study suggested that, at 40 days *post-partum*, the genus *Treponema* was significantly less abundant in the colon and cecum of weaned piglets from the *L. reuteri* group than in those from the chlortetracycline and control groups (Zhang et al., 2016). This is also consistent with the findings of Riboulet-Bisson et al. (2012), who showed that a *Lactobacillus salivarious* strain had the ability to modulate the level of *Treponema*.

It was previously proposed that the major symbiotic function of the gut microbiota is to provide SCFAs by fermenting carbohydrates in the intestinal epithelium, and SCFAs play an important role as an energy source for enterocytes in terms of animal health (Fouhse et al., 2016). Brestensky et al. (2017) reported that, for pigs, the lactate content in the cecal digesta and feces is lower than that in the jejune digesta, whereas acetate is present in higher proportions in the cecal digesta and feces. Liu et al. (2014) found that probiotics could accelerate the decomposition of carbohydrates, influencing SCFAs synthesis and helping to increase the butyric acid concentration in the colon. In the present study, the lactic acid, butyric acid, and the total SCFAs concentrations were all significantly higher whereas the acetic acid level was lower in the *L. reuteri* ZLR003 group compared with the control group. Previous work revealed that acetate provides an important substrate for butyrate production, and about 50% of butyric acid produced by bacteria in the intestine, including *Rosebyria spp*. and *Faecalibacterium prausnitzii*, is associated with the net consumption of acetate (Duncan et al., 2004). Thus, our findings may suggest that dietary supplementation with *L. reuteri* ZLR003 enhanced the production of butyric acid from acetate by the intestinal microbiota.

To date, many studies have used high-throughput sequencing methods to explore the gut microbiota composition of animals. An increasing number of studies have reported the contribution of SCFAs production by the microbiota. Fernandes et al. (2014) reported that SCFAs are absorbed in the colon and that the SCFA content in the feces is lower compared with that in the cecal digesta. Zhang Y.J. et al. (2017) showed that *Lactobacillus* and *Megasphaera* are affected by the fiber source. Louis et al. (2014) reported that *Megasphaera elsdenii* can produce several SCFAs, such as acetate, propionate, butyrate, and valerate. Here, *Lactobacillus* and *Megasphaera* showed positive correlations with butyrate, but there were no significant differences in their abundances between piglets treated by *L. reuteri* and control animals (*P* > 0.05). Additionally, Flint et al. (2008) suggested that the *Ruminococcaceae* genus can produce SCFAs by fermenting dietary polysaccharides in the intestinal tract. However, in the present study, the microbiota results suggest that the abundances of fecal *Ruminococcaceae*_UCG-014 and *Ruminococcaceae*_UCG-010 were significantly lower in the *L. reuteri* group compared with the control group and that *Ruminococcaceae*_UCG-010 and *Ruminococcaceae*_UCG-014 both showed a negative correlation with the acetate and butyrate levels. Thus, it is possible that there is a difference in *Ruminococcaceae* abundance between the intestinal tract and feces, but these differences need to be analyzed in future work.

Here, the *L. reuteri* group showed a higher PUFAs content compared with the control group; this finding is consistent with work by Ross et al. (2012) and Chang et al. (2018). Moreover, an analysis of the LCFAs profiles of piglets also revealed that the PUFAs C18:2n6c, C18:3n3, C20:4n6, and C22:6n3 were significantly higher in the *L. reuteri* ZLR003 group compared with the control group. This is consistent with our finding from a previous study that the PUFAs C18:2n6c, C18:3n3, and C20:4n6 in the colon were significantly higher in the *L. reuteri* group compared with the control group (Zhang D.Y. et al., 2017). Other research has shown that dietary fatty acid composition has an influence on its deposition in tissue, and the PUFAs: SFAs ratio is commonly used to evaluate the nutritional and health value of meat for human consumption (Aldai et al., 2007). Therefore, we hypothesize that *Lactobacillus* may promote the PUFA content in pork during pig production.

Huang et al. (2013) suggested that a diet rich in polyunsaturated omega-6 fatty acids causes an alteration in the composition of the intestinal microbiota compared with diets rich in saturated fat. Mayorga-Reyes et al. (2016) also suggested that a diet rich in unsaturated fatty acids promotes an abundance of beneficial bacteria such as *Bacteroidetes*. *Prevotella*, a dominant genus of *Bacteroidetes*, has been reported to be a polysaccharide-degrading and oligosaccharide-utilizing bacterial genus, associated with the ability to degrade mucin and plant-based carbohydrates (Lamendella et al., 2011; Patel et al., 2014). Previous research showed that *Prevotella* is one of the abundant genera in pigs after weaning and that it represents up to 30% of all classifiable bacteria at 10 weeks of age while accounting for only 3.5–4.0% of the bacteria by the time pigs reach 22 weeks old (Isaacson and Kim, 2012; Pajarillo et al., 2014). Furthermore, the administration of *Lactobacillus* strains was found to significantly increase the abundance of the *Prevotella* genus (Kaluzna-Czaplinska et al., 2017). Our present study found that the genus *Prevotella*, especially *Prevotella*_9, was more abundant in the *L. reuteri* group compared with the control group. Moreover, our results also demonstrate that *Prevotella*_9 showed a stronger positive correlation with C18:2n6c compared with the correlation between the *Prevotella* genus and the SCFAs content.

The serum concentration of FAAs is affected by their intestinal absorption, transport rates across tissue membranes, and cellular metabolism, and this concentration is an important modulator of host physiology (Bertolo et al., 2000). A previous study focused on frail older patients suggested that altered amino acid metabolism is correlated with body composition and nutritional status; it also reported that body mass index was reduced in the severely frail group and that it was significantly correlated with plasma Val levels (Adachi et al., 2018). Essen-Gustavsson et al. (2010) evaluated horses during short and intense exercise (mostly anaerobic) on a high-speed treadmill and reported that there was an interaction between exercise and dietary crude protein in terms of the Lys and Val plasma concentrations. Here, we analyzed the effect of dietary supplementation with the *L. reuteri* ZLR003 strain on the serum FAA profiles of weaned piglets, and our results show that FAAs, including Gly, Ala, Val, Iso, Asp, Asn, Glu, Met, Phe, and Leu, were significantly abundant in the *L. reuteri* group compared with the control group. Chang et al. (2018) reported that muscles from the probiotic-treated group had higher levels of Gly, Ala, Pro, Val, Leu, and Iso. However, intervention studies of amino acids and their relationship with probiotic strains in pig production are still needed.

Lastly, our present study found that the genera *Treponema_2, Lachnospiraceae_XPB1014_group, Clostridium_sensu_stricto_1*, and *Mollicutes_RF9_norank* were negatively correlated with Pro, Asp, Phe, Arg, Met, Tyr, Iso, His, and Leu and that *Streptococcus* was negatively correlated with Phe, Tyr, Met, and Leu, whereas the genera *Megasphaera* and *Mitsuokella* were positively correlated with Pro, Phe, Met, Tyr, Iso, and Leu. The microbiota results also show that dietary *L. reuteri* supplementation significantly increased the abundance of *Megasphaera* and *Mitsuokella* and decreased the abundance of *Streptococcus*. *Mitsuokella* genera, such as *Mitsuokella jalaludinii*, which are considered novel phytase-producing rumen bacteria, were reported to promote the digestion of amino acids (Lan et al., 2011). *M. elsdenii* has been reported as a major inhabitant of the pig intestine, and this bacterium converts lactate to various kinds of SCFAs (Kim et al., 2011), although its relationship with amino acid fermentation is still unclear. Anhe et al. (2015) reported that the main SCFAs produced by the microbiota, such as acetate, propionate, and butyrate, are derived from both carbohydrates and amino acids. Therefore, further investigations are needed to clarify the mechanism of action and obtain a more detailed understanding of the role of gut microbiota composition in nutrient metabolism in pig production.

In conclusion, the results of this study indicate that dietary supplementation with *L. reuteri* ZLR003 in piglets influenced the fecal microbiota composition, which was associated with the metabolism of SCFAs in the feces and the metabolism of LCFAs and FAAs in the serum. This is the first study to analyze the microbiota associated with host metabolism in weaned piglets after the administration of oral *Lactobacillus*. Although further studies are needed, our findings will facilitate the application of *Lactobacillus* strains for nutritional and health benefits in pig production.

## Ethics Statement

The study was carried out in accordance with the recommendations of guidelines set by the Animal Care and Use Committee (permit number: SYXK-2017-0005) of the Institute of Animal Husbandry and Veterinary Medicine, Beijing Academy of Agriculture and Forestry Sciences (IAHVM-BAAFS), Beijing, China. The protocols were approved by the Animal Care and Use Committee of IAHVM-BAAFS.

## Author Contributions

DZ and HJ designed the experiments and carried all the analyses, interpreted the results, and drafted the manuscript. HL contributed to data collection during the animal experiments. SW analyzed the microbiota composition data. WZ measured and analyzed the serum amino acids contents. JW, HT, and YW collected the samples during the animal experiments. All co-authors provided their comments, have read, and approved the final manuscript.

## Conflict of Interest Statement

The authors declare that the research was conducted in the absence of any commercial or financial relationships that could be construed as a potential conflict of interest.

## Footnotes

1 http://www.cnfood.cn/shendubaodao95182.html

2 http://drive5.com/uparse/

3 http://rdp.cme.msu.edu/

4 www.i-sanger.com

## Supplementary Material

FIGURE S1 Rarefaction curves based on the 16S rRNA sequencing in nursed piglets. C, control group; L, *L. reuteri* ZLR003 group.

FIGURE S2 Rarefaction curves based on the 16S rRNA sequencing in weaned piglets. C, control group. L, *L. reuteri* ZLR003 group.

TABLE S1 Sample collection method for piglets.
