# Supplementary material for: Fecal Microbiota and Its Correlation With Fatty Acids and Free Amino Acids Metabolism in Piglets After a Lactobacillus Strain Oral Administration
Source: Front Microbiol. 2019 Apr 16;10:785. doi: 10.3389/fmicb.2019.00785 (PMC6476935; doi:10.3389/fmicb.2019.00785)
Supplement: TABLE S1 — Sample collection method for piglets. [file Table_1.docx]

Supplementary Table S1. Sample collection method for piglets.

| Time | Control group | | | *Lactobacilus* group | | |
| --- | --- | --- | --- | --- | --- | --- |
|  | samples | piglets | items | samples | piglets | items |
| Day 30 | feces | 4 | microbiota | feces | 4 | microbiota |
| Day 60 | feces | 6 | microbiota | feces | 6 | microbiota |
|  |  | 6 | SCFAs |  | 6 | SCFAs |
|  | serum | 8 | LCSAs | serum | 8 | LCSAs |
|  |  | 8 | FAAs |  | 8 | FAAs |
